# Excessive Sensory Stimulation during Development Alters Neural Plasticity and Vulnerability to Cocaine in Mice

**DOI:** 10.1523/ENEURO.0199-16.2016

**Published:** 2016-08-23

**Authors:** Shilpa Ravinder, Elizabeth A. Donckels, Julian S. B. Ramirez, Dimitri A. Christakis, Jan-Marino Ramirez, Susan M. Ferguson

**Affiliations:** 1 Center for Integrative Brain Research, Seattle Children’s Research Institute, Seattle, Washington 98101; 2 Center for Child Health, Behavior and Development, Seattle Children’s Research Institute, Seattle, Washington 98121; 3 Department of Pediatrics, University of Washington, Seattle, Washington 98195; 4 Department of Neurological Surgery, University of Washington, Seattle, Washington 98195; 5 Department of Psychiatry and Behavioral Sciences, University of Washington, Seattle, Washington 98195

**Keywords:** amygdala, drug addiction, environment, mice, nucleus accumbens, sensory stimulation

## Abstract

Early life experiences affect the formation of neuronal networks, which can have a profound impact on brain function and behavior later in life. Previous work has shown that mice exposed to excessive sensory stimulation during development are hyperactive and novelty seeking, and display impaired cognition compared with controls. In this study, we addressed the issue of whether excessive sensory stimulation during development could alter behaviors related to addiction and underlying circuitry in CD-1 mice. We found that the reinforcing properties of cocaine were significantly enhanced in mice exposed to excessive sensory stimulation. Moreover, although these mice displayed hyperactivity that became more pronounced over time, they showed impaired persistence of cocaine-induced locomotor sensitization. These behavioral effects were associated with alterations in glutamatergic transmission in the nucleus accumbens and amygdala. Together, these findings suggest that excessive sensory stimulation in early life significantly alters drug reward and the neural circuits that regulate addiction and attention deficit hyperactivity. These observations highlight the consequences of early life experiences and may have important implications for children growing up in today’s complex technological environment.

## Significance Statement

Environmental stimulation in the form of enrichment has been shown to be beneficial for brain development and behavior. Although this has been broadly interpreted as stimulating the developing brain is advantageous, recent work demonstrates that sensory stimulation can in fact have negative consequences, particularly if it is non-normative and extensive, and is presented during development. This research adds to existing knowledge on the impact of early life experiences and provides fundamental insights into how environmental factors during development can shape the brain and behavior. At a point where childhood and adolescence is increasingly dominated by exposure to audiovisual media, we believe our findings build the case for further investigation into the effects of extended exposure to sensory experiences in early life.

## Introduction

Attention-deficit/hyperactivity disorder (ADHD) and drug addiction are neuropsychiatric diseases with a high comorbidity rate and a strong genetic component (Capusan et al., 2016). However, there remains a large role for environmental factors in the etiology of these diseases (McCrory and Mayes, 2015). It is widely recognized that early life experiences shape neural function, which can have lasting impacts on behavior and result in vulnerability to the development of these diseases. For example, childhood stress during periods of critical development increases the propensity to impulsive choice, ADHD, and drug use/abuse later in life, whereas positive life experiences, such as good family and peer relations, can be protective against the development of ADHD and decrease the likelihood of drug use (Jessor and Jessor, 1980; Kodjo and Klein, 2002; Sinha, 2008; Enoch, 2012). Studies in rodent models have found similar effects. Animals exposed to stress early in life show impulsivity, impaired decision-making, greater motivation to seek drugs, and increased rates of drug-induced reinstatement (McEwen, 2003; Rüedi-Bettschen et al., 2006; Andersen and Teicher, 2009). On the other hand, rodents reared in an enriched environment, which provides plenty of complex inanimate and social stimulation, have enhanced decision-making and cognition, decreased motivation to seek drugs, and lower rates of drug-induced reinstatement (Solinas et al., 2010; Takuma et al., 2011).

Although much of the laboratory animal work on environmental risk factors has focused on impoverished versus enriched environments, recent studies in humans have shown that exposure to extensive periods of auditory and visual stimulation during childhood is highly correlated with attentional problems (Christakis et al., 2004; Zimmerman and Christakis, 2007). However, human studies cannot be used to establish a causal relationship between excessive sensory exposure and behavioral consequences. As such, we have only a limited understanding of how increased sensory stimulation alters brain function and behavior, and changes the risk for neuropsychiatric illness. While the introduction of animal models to study the consequences of an enriched environment has led to deep and detailed insights into the underlying cellular mechanisms, we know very little about the consequences of excessive sensory stimulation (ESS). Only two recent studies have investigated the effects of repetitive sensory stimulation. One study (Hadas et al., 2016) showed that repetitive olfactory stimulation during development in rats impaired performance in an attention task in the presence of an auditory distractor. Using repetitive auditory and visual stimulation in a mouse model, the second study (Christakis et al., 2012) reported that extended exposure to sensory stimulation during development produces pronounced hyperactivity, impaired cognition, and increased novelty seeking. In the present study, we have used the same mouse model to examine the effects of excessive exposure to sensory stimulation during development on the rewarding and psychomotor activating effects of cocaine, using conditioned place preference and locomotor sensitization, respectively. In addition, we characterized whether this stimulation protocol produces baseline changes in neural activity in two components of the neural circuits thought to contribute to addiction and ADHD, the nucleus accumbens (NAc) and the amygdala.

## Materials and Methods

### Experimental animals

Male CD-1 mice purchased from Charles River Laboratories (RRID:SCR_013551) were used for all experiments. Mice (postweaning) were group housed (three to five per cage) with *ad libitum* access to food and water under a 12 h light/dark cycle (light on at 7:00 A.M.) and with a controlled temperature (22 ± 1°C). All experiments and animal procedures were performed in accordance with the regulations of the Seattle Children’s Research Institute animal care committee, and were conducted in accordance with the National Institutes of Health guidelines.

### Excessive sensory stimulation paradigm

Mice received sensory stimulation in their home cages for 42 consecutive days starting at postnatal day 10 (P10). The stimulation occurred during the dark cycle for 6 h/d. The dam was stimulated along with the pups from P10 until weaning (P21). Control groups were raised under standard laboratory housing conditions and tested at corresponding times with the sensory stimulation groups. The sensory stimulation setup consisted of two loudspeakers, suspended 2 inches above the top of the cage. Auditory stimulation consisted of audio from television cartoon shows (e.g., Pokemon, Powerpuff girls, Bakugan), which were layered on top of each other, with one pitch shifted (10–20 kHz), and one non-pitch-shifted track in order to better accommodate the higher-frequency hearing range of mice. Sounds were no louder than 70 dB, which is significantly lower than the common auditory stress model. Light-emitting diode (LED) lights (red, green, yellow, and blue) were placed around the cages to provide visual stimulation. A photorhythmic modulator was used to change the frequency of the blinking LED lights in concordance with the sound output from the speakers.

### Behavioral tests

#### Conditioned place preference

The conditioned place preference (CPP) test is a classic pavlovian conditioning procedure used to study the reinforcing effects of unconditioned stimuli (e.g., drugs, food). The CPP test was performed in a three-chamber place-preference box (ENV-3013, Med Associates) using an unbiased, three-phase design (preconditioning, conditioning, and postconditioning). The CPP test was conducted on control and ESS mice from P52 to P56 (preconditioning test, P52; conditioning, P53 to P55; postconditioning test, P56). The apparatus consisted of two large compartments separated by a central neutral compartment. The two lateral compartments differed in floor texture and wall pattern: vertically striped walls and stainless steel grid rods for flooring on one side, and horizontally striped walls and metal mesh flooring on the other; the small central compartment had a smooth floor. During the preconditioning phase, mice were placed in the central compartment and allowed 15 min of free access to all compartments of the CPP box. During the conditioning phase, mice received twice daily (morning and afternoon) conditioning sessions for 3 d. On each conditioning day, mice were confined to one compartment for 15 min immediately following the administration of saline (morning) or cocaine (15 mg/kg, i.p.; obtained from the National Institute on Drug Abuse; afternoon). The choice of compartment for saline/cocaine pairing was randomized and counterbalanced across groups. During the postconditioning phase, mice were given 15 min of free access to the CPP apparatus on the day following the final conditioning session. Time spent in the compartments was tracked using Noldus EthoVision XT 8.0. A CPP score was calculated for each mouse as the difference between preconditioning and postconditioning time spent in the drug-paired compartment. A change in preference for the drug-paired compartment serves as an index of the reinforcing effects of cocaine.

#### Activity assessment and psychomotor sensitization

Activity levels in mice and the psychomotor activating effects of cocaine were measured using locomotor activity boxes (8.5 × 17.5 × 9 inches) from San Diego Instruments (SDI) that contained regular ground corncob bedding on the floor. The Photobeam Activity System software (SDI) was used to track total crossovers in a 4 × 8 photobeam configuration, which provided a measure of locomotor activity. To induce psychomotor sensitization, mice received 10 treatment sessions over a 2 week period (induction phase, P52–P65). During each session, mice were habituated to the locomotor chambers for 45 min followed by an injection of cocaine (15 mg/kg, i.p.) or saline, and locomotor activity was monitored for 60 min. After a 2 week withdrawal period, all mice received an escalating dose challenge of cocaine (challenge phase). During this phase, mice received a 45 min habituation period, followed by sequential injections of saline 10 mg/kg and cocaine 20 mg/kg spaced 60 min apart. Locomotor activity was monitored for the entire duration of the session, and total crossovers within the 60 min sessions were plotted and used for statistical analysis.

### *In vitro* slice electrophysiology

Slice electrophysiology experiments were conducted on control and ESS mice at P52–P70. Their brains were quickly removed under deep anesthesia, and 350-μm-thick coronal slices containing the NAc shell or the lateral amygdala (LA) and basal amygdala (BA) were prepared. We chose to study these particular subregions as a vast body of literature shows that these brain regions are interconnected and are required for cocaine-related behaviors, and cellular changes in these subregions are thought to underlie the behavioral effects of cocaine administration (Thomas et al., 2001; Fuchs et al., 2002; Kourrich and Thomas, 2009; Stuber et al., 2011; Lee et al., 2013; Hsiang et al., 2014). Slices were transferred to a submerged chamber containing artificial CSF (aCSF; in mm: 124 NaCl, 2.7 KCl, 26 NaHCO3, 0.4 NaH2PO4, 10 glucose, 4 sodium ascorbate, 1.3 MgCl2, and 2 CaCl2) equilibrated with 95% O_2_/5% CO_2_ at room temperature. Slices were incubated for at least 1 h before being transferred to a superfused recording chamber. Excitatory pyramidal neurons in the BA or medium spiny neurons in the NAc shell were visually identified using a Zeiss Axioskop 2 FS microscope with infrared differential interference contrast. Patch electrodes (3–6 MΩ) were pulled from borosilicate glass pipettes on a P-97 Flaming-Brown Micropipette Puller (Sutter Instruments) and filled with the voltage-clamp pipette internal solution [for miniature EPSCs (mEPSCs; in mm): 120 CsOH, 120 gluconic acid, 20 CsCl, 10 HEPES, 4 MgATP, and 0.3 NaGTP, and 10 phosphocreatine, pH 7.3, 300 mOsm; for mIPSCs (in mm): 140 CsCl, 10 10 HEPES, 10 phosphocreatine, 4 MgATP, and 0.3 NaGTP, pH 7.3, 290 mOsm]. Whole-cell patch-clamp recordings were performed using an Axon Multiclamp 700B patch-clamp amplifier. All recordings were performed at 30°C. Neurons were voltage clamped at −70 mV. mEPSCs were isolated by using 75 μm picrotoxin and 0.5 μm TTX in the aCSF solution, and miniature IPSCs (mIPSCs) were isolated by adding 10 μm CNQX, 30 μm d-APV, and 0.5 μm TTX in the aCSF solution. Continuous current traces were recorded for a 5 min period. Series resistance (R_s_) was monitored before and after the experiment, and only cells with an R_s_ value <25 MΩ were taken for analysis. Data were filtered at 2.1 kHz and digitized at 10 kHz. The amplitude and frequency of mEPSCs and mIPSCs were analyzed using the Mini Analysis Program (Synaptosoft). The firing output of BA neurons was measured in the current-clamp mode [internal solution composition (in mm): 140 K-gluconate, 10 HEPES, 1 CaCl_2_, 2 MgSO_4,_ 4 Na_2_ATP, 0.3 Na_2_GTP, and 10 EGTA], and the membrane potential was adjusted to −70 mV before the injection of each current pulse. Action potential firing in response to a series of depolarizing current steps was recorded. Saturating current intensities were excluded from the analysis. Some basic properties of BA principal neurons were also measured in the current-clamp mode. The resting membrane potential (*V*_m_) was measured immediately after achieving whole-cell configuration by bringing the holding current to 0 pA. The action potential threshold was estimated by injecting a ramp of current (0–500 pA in 100 ms) and measuring the voltage at which the first action potential occurred. The current–voltage relationship (*I–V* curve) was analyzed by measuring the peak voltage response to a series of current steps ranging from −100 to 50 pA. The input resistance was calculated as the slope of the *I–V* curve for each neuron.

### Corticosterone measurement

Plasma corticosterone (CORT) levels in control mice and mice that received sensory stimulation were quantified using an ELISA. Following 42 d of the sensory stimulation protocol, at age P52, mice were killed and blood samples were collected for CORT measurements. Mice were anesthetized with isoflurane and decapitated to collect trunk blood into lithium heparinized tubes (microcontainer 365971, BD). The blood samples were then centrifuged at 10,500 rpm for 10 min at 4°C to isolate plasma. The supernatant was then collected into Eppendorf tubes and stored at −80°C until further analysis. To quantify CORT levels, the plasma sample were thawed, and ELISA assays were performed by following the manufacturer instructions [catalog #KO14-H5, Arbor Assays (RRID:SCR_013534)].

### Statistical analyses

Statistical analyses were conducted using either two-way repeated-measures ANOVA with Bonferroni’s *post hoc* analysis (to correct for multiple comparisons), or a one-sample or two-sample *t* test (without correction) when appropriate and as indicated, using Prism (GraphPad; RRID:SCR_000306). Differences were considered to be statistically significant at *p* ≤ 0.05.

## Results

### Exposure to excessive sensory stimulation during development enhances CPP to cocaine

**Figure 1.**
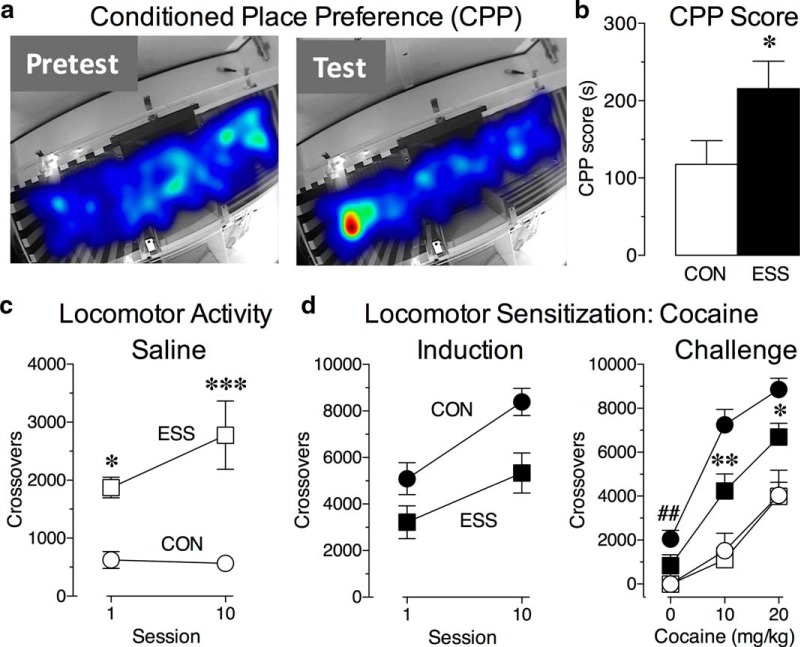
Exposure to excessive sensory stimulation (ESS) during development alters behavioral responses to cocaine and locomotor activity. ***a***, Representative heat map of time spent in the different compartments of the CPP box during the pretest (left) and on the test (right). ***b***, Mice exposed to ESS during development had a significantly greater CPP score compared with control (CON) mice (*p < 0.05 vs CON; *n* = 13-14/group). ***c***, Locomotor activity following saline administration in CON mice (white circles) and ESS (white squares) mice, as measured by the total number of crossovers. Exposure to ESS during development led to a significant increase in locomotion compared with controls (*p < 0.05 vs CON mice in session 1; ***p < 0.001 vs CON mice in session 10; *n* = 7-9/group). ***d***, Left, induction phase, Total number of crossovers made during the 60 min following cocaine injection normalized to baseline responding (i.e., the average total crossovers in the corresponding saline group were subtracted from total crossovers for each mouse) in CON (black circles) and ESS (black squares) mice. Exposure to ESS during development had no effect on the development of locomotor sensitization during cocaine treatment (*n* = 10-11 mice/group). Right, challenge phase, Total number of crossovers made during the 60 min following each dose of a multidose challenge (0, 10, and 20 mg/kg cocaine). Responses normalized to the corresponding saline pretreatment group at the 0 mg/kg challenge. Control mice that received cocaine during the induction phase, but not mice that were exposed to ESS during development, displayed a conditioned locomotor response (**##***p* = 0.009 vs saline-pretreated CON mice). In addition, ESS mice showed a significantly blunted locomotor sensitization to cocaine (**p = 0.007 vs cocaine-pretreated CON; *p = 0.05 vs cocaine-pretreated CON mice in session 10; *n* = 7-11 mice/group). Data represent the mean ± SEM.

The rewarding effects of cocaine were assessed in controls and mice that received ESS using a CPP procedure; testing was performed in a drug-free state (Fig. 1*a*). We found that both groups of mice acquired a clear preference for the cocaine-paired chamber (Fig. 1*b*; CON: *t*_(13)_ = 3.81, *p* = 0.002; ESS: *t*_(12)_ = 6.08, *p* < 0.0001). However, mice that received sensory stimulation during development had a significantly greater CPP score compared with controls (Fig. 1*b*; *t*_(25)_ = 2.09, *p* = 0.04), suggesting that they had a more robust response to the rewarding properties of cocaine.

### Exposure to excessive sensory stimulation during development impairs the persistence of cocaine-induced locomotor sensitization

In a separate cohort of mice, we assessed locomotor activity and the development of cocaine sensitization in control and ESS mice. As expected from other behavioral tests (Christakis et al., 2012), mice that received sensory stimulation during development were significantly more active than controls on the first day of saline treatment, and this effect was stronger by the last test session [Fig. 1*c*; main effect of stimulation: *F*_(1,14)_ = 18.02, *p* = 0.0008; *p* < 0.05 (session 1) and *p* < 0.001 (session 10) vs control]. Thus, sensory stimulation during development led to hyperactivity that became increasingly more pronounced with repeated exposure to the testing environment. Given the differences in locomotor activity in saline groups, the responses of the cocaine groups were normalized to these different baselines (by subtracting the average total crossovers in the corresponding saline group from the total crossovers for each mouse) in order to gain a clearer picture of the impact of developmental sensory stimulation exposure on locomotor sensitization to cocaine. During the induction phase of sensitization, we found that the acute locomotor response to cocaine was decreased in mice that received extended periods of sensory stimulation during development compared with controls, although this effect did not quite reach statistical significance [Fig. 1*d*, left; main effect of stimulation: *F*_(1,19)_ = 10.82, *p* = 0.004; *p* = 0.1 (session 1) vs control]. Nonetheless, both groups showed significant increases in locomotor responses following repeated cocaine treatment, suggesting that sensitization had developed in all mice (Fig. 1*d*, left; main effect of session: *F*_(1,19)_ = 14.97, *p* = 0.001; no interaction: *F*_(1,19)_ = 0.71, *p* = 0.41).

Following a 2 week withdrawal period, all mice underwent a challenge session, which included an injection of saline to test for the development of a conditioned response in mice that had previously received cocaine injections. As expected, control mice showed a conditioned locomotor response to this saline injection; however, mice that were exposed to the sensory stimulation protocol did not (Fig. 1*d*, left; main effect of pretreatment: *F*_(1,33)_ = 10.17, *p* = 0.003; *p* = 0.009 vs saline-pretreated control). In addition, both groups of mice that received cocaine treatment during the induction phase showed greater locomotor responses to the challenge doses of cocaine compared with the saline-treated mice. However, the cocaine-treated mice that received the sensory stimulation exposure during development had significantly decreased locomotor responses during the cocaine challenge compared with controls (Fig. 1*d*, right; 10 mg/kg: main effect of pretreatment: *F*_(1,33)_ = 36.47, *p* < 0.0001; main effect of stimulation: *F*_(1,33)_ = 5.42, *p* = 0.03; *p* = 0.007 vs cocaine-pretreated controls; 20 mg/kg: main effect of pretreatment: *F*_(1,33)_ = 27.57, *p* < 0.0001; *p* = 0.05 vs cocaine-pretreated controls), suggesting that the persistence of sensitization was impaired. Thus, despite the fact that exposure to excessive sensory stimulation during development produces hyperactivity, it also results in blunted locomotor sensitization to cocaine.

### Exposure to excessive sensory stimulation is not stressful

**Figure 2.**
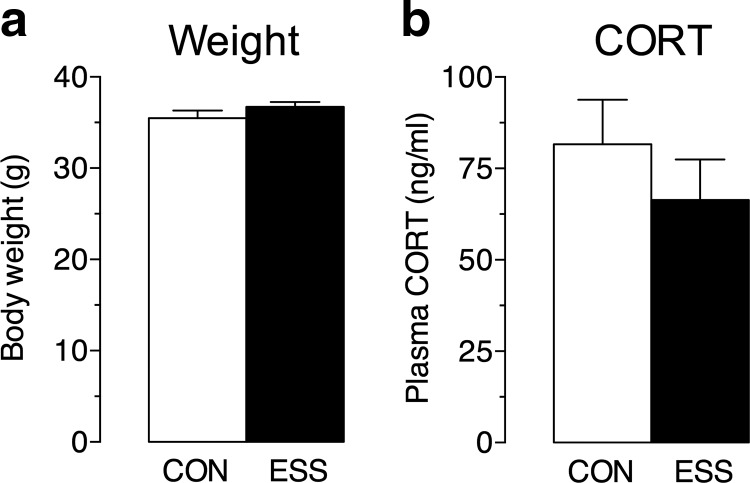
Exposure to excessive sensory stimulation (ESS) does not affect measures of a stress response. ***a***, Exposure to ESS during development does not alter body weight at P53 (i.e., the day after the end of ESS exposure) compared with control (CON) mice (*n* = 32-39 mice/group). ***b***, Plasma CORT levels at P53. Exposure to ESS during development does not affect baseline plasma CORT levels compared with CON mice (*n* = 10 mice/group). Data represent the mean ± SEM.

Stress is a well known modulator of the behavioral effects of cocaine (Shaham et al., 2000; Kreibich et al., 2009). Thus, to assess whether the stimulation paradigm results in a stress phenotype, body weights and plasma CORT levels were measured in mice at P53 (i.e., 24 h following the last stimulation exposure). We found that body weights were the same in mice that were exposed to excessive periods of sensory stimulation during development and in control mice (Fig. 2*a*; *t*_(69)_ = 1.30, *p* = 0.20). In addition, there were no differences in plasma CORT levels between control mice and those that underwent the sensory stimulation protocol (Fig. 2*b*; *t*_(18)_ = 0.93, *p* = 0.37). These observations suggest that the extended exposure to lights and sounds used in the sensory stimulation protocol does not alter baseline stress levels in the mice.

### Exposure to excessive sensory stimulation during development increases the frequency of miniature EPSCs in limbic circuits

**Figure 3.**
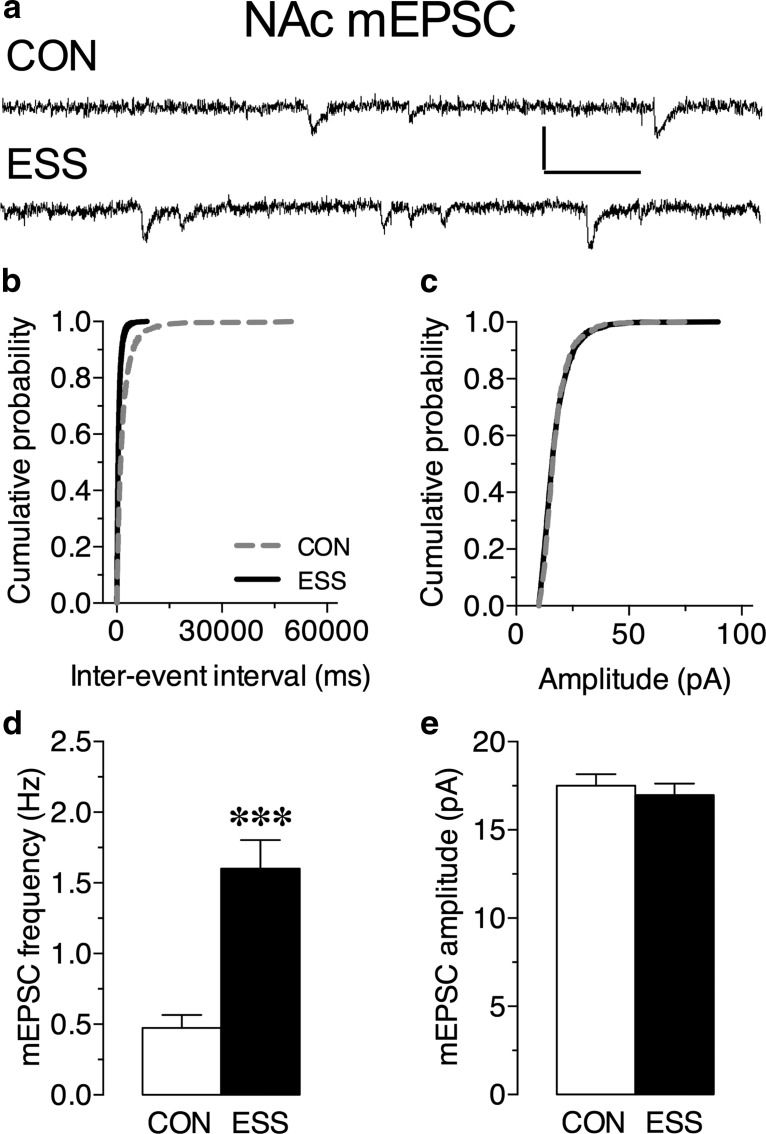
Excessive sensory stimulation (ESS) ESS enhances excitatory tone in the nucleus accumbens (NAc) shell. ***a***, Representative mEPSC traces from NAc shell neurons in slices from control (CON) and ESS mice. ***b***, ***c***, Cumulative probability distribution for interevent interval (***b***) and amplitude (***c***) of mEPSCs in NAc shell neurons. ***d***, ***e***, Exposure to ESS during development significantly increased the frequency (***p = 0.0002; *n* = 9-11 cells/group; *N* = 3-4 mice/group), but not the amplitude of mEPSCs in the NAc shell compared with CON mice. Calibration: 20 pA (vertical axis), 50 ms (horizontal axis). Data represent the mean ± SEM.

To begin to explore neural correlates of the observed behavioral changes, we next examined whether exposure to excessive sensory stimulation produces a fundamental shift in neuronal activity by measuring mEPSCs in the shell region of the NAc, as well as the LA and BA nuclei of the amygdala. In the NAc (Fig. 3), we found that, while mEPSC amplitude was not different between groups (Fig. 3*e*; *t*_(18)_ = 0.57, *p* = 0.58), there was a significant increase in the frequency of mEPSCs in the mice that received sensory stimulation during development compared with control mice (Fig. 3*d*; *t*_(18)_ = 4.67, *p* = 0.0002).

**Figure 4.**
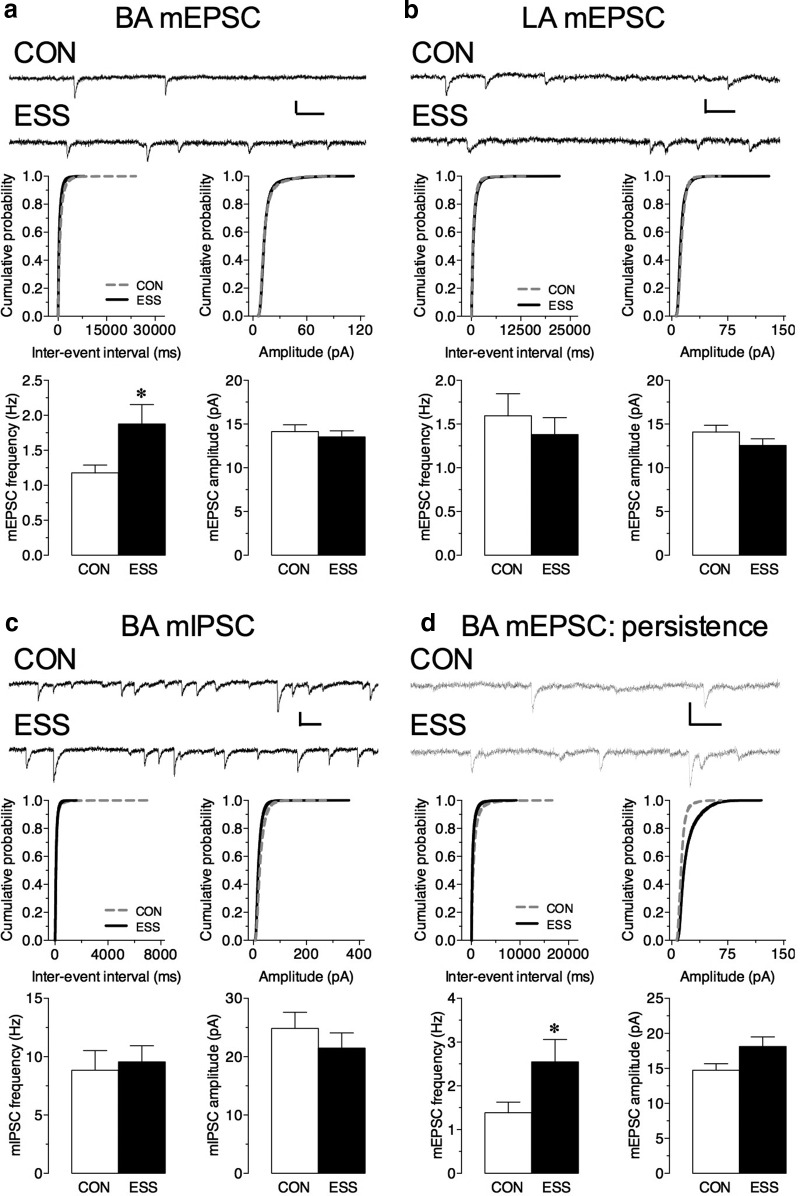
Excessive sensory stimulation (ESS) during development enhances excitatory tone in the basal amygdala (BA). ***a***, ***b***, Top, Representative mEPSC traces from BA (***a***) and lateral amygdala (LA) (***b***) principal neurons in slices from ESS and control (CON) mice. Bottom, Exposure to ESS during development significantly increased the frequency of mEPSCs in the BA (***a***, left: *p = 0.03; *n* = 16-17 cells/group; *N* = 4-9 mice/group) but not in the LA (***b***, left: *n* = 10 cells/group; *N* = 3-4 mice/group) compared with CON mice. There was no effect of this manipulation during development on the amplitude of mEPSCs in the BA (***a***, right) or in the LA (***b***, right). Middle, Cumulative probability distribution for interevent interval (left) and amplitude (right) of mEPSCs in BA (***a***, center) and LA (***b***, center) neurons. ***c***, Top, Representative mIPSC traces from BA principal neurons in slices from ESS and CON mice. Bottom, Exposure to ESS during development had no effect on the frequency (left) or the amplitude (right) of mIPSCs in the BA compared to that for CON mice (*n* = 8-10 cells/group; *N* = 3-4 mice/group). Middle, Cumulative probability distribution for the interevent interval (left) and amplitude (right) of mIPSCs in BA neurons. ***d***, Top, Representative mEPSC traces from BA principal neurons in slices from adult ESS and CON mice 2 months after the end of the stimulation protocol. Bottom, The mEPSC frequency (***d***, left: *p = 0.05; *n* = 11-13 cells/group; *N* = 3-4 mice/group) but not amplitude (right) was significantly increased 2 months following the end of ESS. Middle, Cumulative probability distribution for the interevent interval (left) and amplitude (right) of mEPSCs in BA neurons 2 months following the end of ESS. Calibration (***b–e***): 20 pA (vertical axis), 50 ms (horizontal axis). Data represent the mean ± SEM.

Similarly, we found a significant increase in the frequency (*t*_(18)_ = 2.35, *p* = 0.03), but not in the amplitude (*t*_(18)_ = 0.58, *p* = 0.57), of mEPSCs in the BA of young mice that had received sensory stimulation compared with control mice (Fig. 4*a*). In contrast, we found no difference in the frequency (*t*_(18)_ = 0.68, *p* = 0.51) or amplitude (*t*_(18)_ = 1.45, *p* = 0.16) of mEPSCs in the LA (Fig. 4*b*). This observation was specific to excitatory currents in the BA as we observed no difference in either the frequency (*t*_(18)_ = 0.33, *p* = 0.74) or the amplitude (*t*_(18)_ = 0.89, *p* = 0.39) of mIPSCs in BA principal neurons (Fig. 4*c*). Interestingly, we found that the increase in mEPSC frequency (*t*_(17.05)_ = 2.05, *p* = 0.05) in BA neurons persisted even 2 months after the end of stimulation, suggesting that these cellular changes are long lasting (Fig. 4*d*).

**Figure 5.**
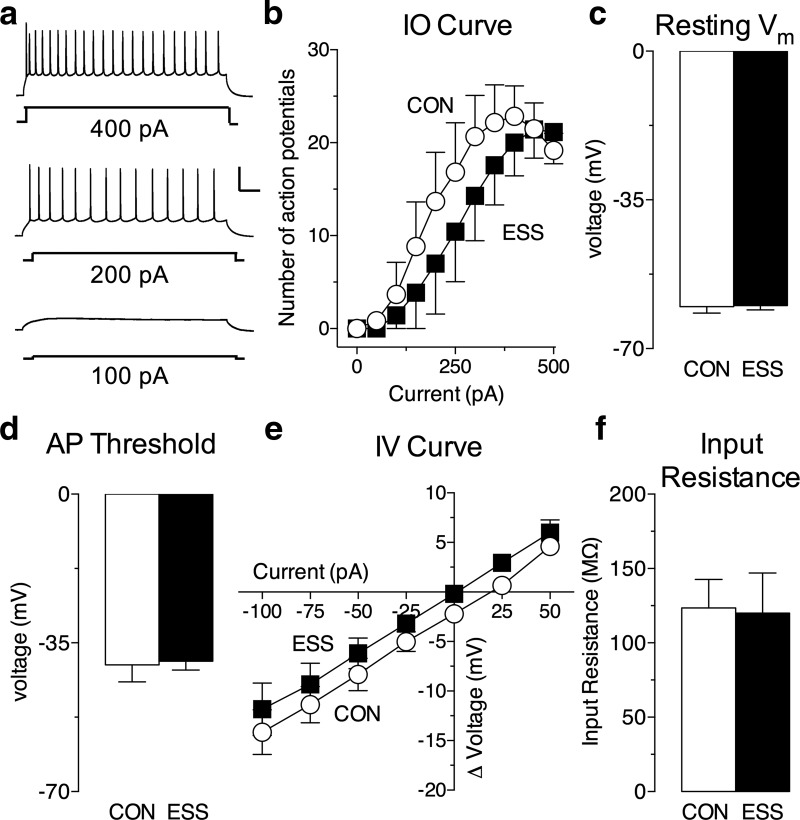
Exposure to excessive sensory stimulation (ESS) does not change action potential firing or basic properties of basal amygdala (BA) principal neurons. ***a***, Representative spike trains evoked by somatic injection of increasing steps of depolarizing currents. ***b***, Input–output (I–O) curve (number of action potentials vs current injected) for BA principal neurons in slices from mice exposed to ESS during development (black squares) and control (CON) mice (white circles). There were no differences in the I–O curve between groups (*n* = 6-7 cells/group; *N* = 3-4 mice/group). ***c–f***, Basic properties of BA principal neurons recorded from control and ESS brain slices. ***c***, Resting *V*_m_ was not different between BA principal neurons in ESS and CON brain slices (*n* = 6-7 cells/group; *N* = 3-4 mice/group). ***d***, Action potential threshold (in millivolts) was not different between BA principal neurons in ESS and CON brain slices (*n* = 6 cells/group; *N* = 3-4 mice/group). ***e***, The *I–V* curve was not different between BA principal neurons in ESS and CON brain slices (*n* = 5-6 cells/group; *N* = 3 mice/group). ***f***, Input resistance was not different between BA principal neurons in ESS and CON brain slices (*n* = 5-6 cells/group; *N* = 3 mice/group). Calibration: 40 mV (vertical axis), 100 ms (horizontal axis). Error bars indicate the mean ± SEM.

In order to test the functional consequence of enhanced mEPSC frequency on BA neurons, we measured the firing output of BA principal neurons. Neurons were current clamped with the membrane potential maintained at −70 mV, and action potential firing in response to somatic injections of increasing steps of depolarizing currents was recorded (Fig. 5*a*). We found that while there was a significant increase in firing rates with current injection across both groups, there was no significant difference in firing rates between cells from slices of mice that received excessive sensory stimulation during development and control mice (Fig. 5*b*; main effect of current: *F*_(10,110)_ = 24.38, *p* < 0.0001; no main effect of stimulation: *F*_(1,11)_ = 0.54, *p* = 0.48). Other basic properties measured in the current-clamp mode, namely, resting membrane potential, action potential threshold, the *I–V* curve, and input resistance, were not different between BA neurons in control and ESS mice (Fig. 5*c–f*). These findings indicate that excessive periods of sensory stimulation lead to a specific increase in the frequency of mEPSCs in the BA and the NAc.

## Discussion

Early-life experiences have critical influences on the development of neural circuits, and on susceptibility to drug use and addiction (Andersen and Teicher, 2009; Solinas et al., 2010). Understanding these influences is very important as early life experiences not only drive adaptation, but, under certain conditions, can be a major source of maladaptation. Enriched environments in rodents are known to be procognitive, to decrease addiction vulnerability, and to enhance brain function, whereas impoverished environments have the opposite effects (Fabel and Kempermann, 2008; Kempermann et al., 2010; Volkers and Scherder, 2011). However, unlike the positive effects of an enriched environment, it has recently been shown that exposure to extended periods of sensory stimulation during development in mice produces ADHD-like symptoms, including hyperactivity, impaired cognition, increased novelty seeking, and increased distractability (Christakis et al., 2012; Hadas et al., 2016). Here we found that exposure to excessive sensory stimulation also enhances the rewarding effects of cocaine while blunting its psychomotor-activating effects. This is a significant finding, given the high comorbidity of ADHD and addiction (Zernicke et al., 2010; Jupp and Dalley, 2014). In addition, this result is consistent with work examining psychostimulant-induced locomotor activity and sensitization using other models of ADHD that express a hyperactive phenotype, such as the dopamine transporter knock-out mouse and the spontaneously hypertensive rat (Sagvolden et al., 2005). However, it is possible that the enhanced CPP observed in the stimulated mice was due to alterations in learning and memory, and this possibility will be explored in future studies.

In addition to these behavioral alterations, this excessive stimulation paradigm leads to a lasting enhancement in the frequency of mEPSCs in principal neurons of the amygdala and NAc, regions that are critical components of the neuronal circuits that regulate cognition, impulsivity, and reward. Although profound and widespread, the neurobiological changes caused by excessive sensory stimulation are very specific. In particular, the baseline increases in mEPSC frequency in the BA and the NAc shell raise the intriguing possibility that excessive sensory experiences during childhood and adolescence lead to a fundamental shift in excitatory drive from sensory inputs to these regions, which in turn could affect the threshold for generating behavioral responses through downstream projections of these regions. Thus, because of an altered set point, children exposed to excessive sensory stimulation may need higher levels of stimulation to elicit a behavioral action, which is reminiscent of children with ADHD. Dissecting the mechanisms underlying these changes, as well as how these alterations in baseline plasticity contribute to the dysregulated behaviors observed in sensory stimulation-induced attentional problems, ADHD, and addiction, warrant future investigation.

The amygdala is an essential component of the circuitry that assigns emotional valence to external stimuli and produces appropriate behavioral responses (Aggleton, 2000; Phelps and LeDoux, 2005). It is also an important part of the brain circuits that regulate learning and memory, anxiety, and addiction (Davis, 1992; Roozendaal et al., 2009; Koob and Volkow, 2010), and aberrant amygdala activity is associated with numerous psychiatric illnesses, including ADHD and addiction (Kilts, 2001; Anand and Shekhar, 2003; King et al., 2003; See et al., 2003). In particular, the BA subregion of the amygdala has been found to play a key role in behaviors related to drug addiction (Baxter and Murray, 2002; Fuchs et al., 2002; Tye and Deisseroth, 2012; Heldt et al., 2014; Hsiang et al., 2014). Similarly, the NAc is also a critical component of these circuits, and changes in NAc activity are also associated with ADHD and addiction (Genro et al., 2010; Koob and Volkow, 2010). Specifically, the integration of dopaminergic reinforcement signals with glutamatergic signals (from the amygdala, hippocampus, medial prefrontal cortex, and thalamus) that encode information about environmental stimuli leads to plasticity in the NAc that is thought to underlie motivation, reward, and drug-taking and drug-seeking behaviors (Yager et al., 2015). Further, the shell region of the NAc is particularly important for mediating both the rewarding and psychomotor-activating effects of cocaine (Pontieri et al., 1994, 1995; Caine et al., 1995; Pierce and Kalivas, 1995; McKinzie et al., 1999; Parkinson et al., 1999). Given that the BA and NAc are interconnected and can influence circuit function and plasticity, it is likely that the electrophysiological changes that occur in these brain regions following excessive sensory stimulation are contributing to the altered behavioral responses to cocaine (Stuber et al., 2011; Britt et al., 2012; MacAskill et al., 2014).

The sensory stimulation paradigm used in the present set of experiments does not appear to be inherently stressful to mice. The audio stimulation in this model (70 dB) is well below the levels typically used in acoustic stress models (100–115 dB). Moreover, stress leads to an increase in anxiety-like behavior (Conrad et al., 1999; Vyas and Chattarji, 2004), while a previous report (Christakis et al., 2012) has found that young mice receiving excessive periods of sensory stimulation show a decrease in anxiety-like behavior. Stress can also affect body weight gain (Vyas et al., 2002; Gao et al., 2011); however, we found no difference in body weights between controls and mice exposed to sensory stimulation (Fig. 3*a*). In addition, repeated exposure to a stressor normally triggers a hypothalamic–pituitary–adrenal axis response, leading to alterations in baseline plasma CORT levels (Odio and Brodish, 1989); yet, we found that baseline plasma CORT levels in mice that underwent the sensory stimulation protocol were comparable to those in controls (Fig. 3*b*). Thus, there is no indication that the neurobiological and behavioral effects reported here are caused by stress, experienced directly or indirectly via maternal stress.

Understanding the impact of excessive exposure to sensory stimulation is highly relevant to today’s society. Although animal models do not use the type of stimuli that rodents typically encounter under natural circumstances and cannot fully mimic the human experience, they have nonetheless contributed to a deep mechanistic understanding of the effects of environmental enrichment. Yet we have only very limited mechanistic insights into the consequences of exposure to sensory hyperstimulation. Here we show that in the developing brain that excessive exposure to auditory and visual stimulation alters behavioral susceptibility to cocaine and changes baseline neuronal activity in associated neural circuits. It is conceivable that abnormally patterned stimulation or even too much sensory stimulation may contribute to the rise in ADHD diagnoses that have started occurring in the past decade, which could in turn influence addiction rates. Interestingly, our research findings along with previous studies on sensory stimulation in rodents are reminiscent of clinical observations in children exposed to extensive television viewing and resemble the three core clinical dimensions of ADHD (inattentiveness, impulsivity, and hyperactivity). In addition, stimulants such as Ritalin normalize the hyperactivity associated with ADHD, and, consistent with this, we found that cocaine-induced locomotor sensitization was blunted in mice that received extended periods of sensory stimulation. Finally, children with ADHD have an increased risk for the development of drug abuse and addiction (Harstad and Levy, 2014), and we found that mice that received sensory stimulation displayed an increase in the rewarding effects of cocaine, indicating an enhanced vulnerability to drugs of abuse. Thus, the excessive sensory stimulation paradigm provides a highly relevant model to understand how environments that contain excessive and ill-patterned stimuli influence behavioral outcomes, change neuroplasticity, and influence the propensity to the development of neuropsychiatric disorders, such as ADHD and addiction.

## Synthesis

The decision was a result of the Reviewing Editor Karen Szumlinski and the peer reviewers coming together and discussing their recommendations until a consensus was reached. A fact-based synthesis statement explaining their decision and outlining what is needed to prepare a revision is listed below. The following reviewers agreed to reveal their identity: Carrie Ferrario, Jeffrey Grimm

Synthesis: Two of the previous reviewers of your article, originally submitted to Neuropsychopharmacology, have now reviewed the revised version. In consultation with these reviewers, we have reached the consensus that your report entitled: "Excessive sensory stimulation during development alters neural plasticity and vulnerability to cocaine in mice", should be acceptable for publication in eNeuro pending address of some remaining issues raised by Reviewer #1. All three of us agree that the authors have worked hard to address the concerns of the original reviewers of the article but the remaining major concern regarding the use of Bonferroni corrections for the multiple t-tests must be addressed in order to proceed with Editorial review and article acceptance.

Reviewer #1:

The revised manuscript entitled, "Excessive sensory stimulation during development alters neural plasticity and vulnerability to cocaine in mice." has been substantially improved by the authors. The presentation of the methods is much clearer overall, and the authors were very responsive to several comments to "tone down" the link to human sensory experience during adolescence. In addition, the discussion of the potential role of stress is clear, concise, and reasonable based on the current available data.

Overall, this is a nice set of studies that brings a new approach to examining long-term consequences of early life sensory experience on brain and behavior. I have only a few additional comments that can be easily addressed (see below).

Major Comments:

1) In regards to statistics, my original question regarding correcting for the number of comparisons still stands. From the methods: "Statistical analyses were conducted using either ANOVA with Bonferroni's post hoc analysis (to correct for multiple comparisons) or a one-sample or two-sample t test when appropriate and as indicated, using GraphPad Prism (GraphPad Software)." From this statement, it seems that no correction of the number of one- and two-sample t-tests were used. If this is not the case, please specify that Bonferroni (or other) correction measures were used for all comparisons. If, however, the number of t-tests run (more than 5 in total) were not corrected for, then this should be stated separately.

2) Regarding sensitization, the authors state, "In addition, although both groups showed significant increases in locomotor responses following repeated cocaine treatment, mice that were exposed to the sensory stimulation protocol had significantly lower locomotor responses to cocaine on session 10 compared to controls (Fig. 1d, left; P = 183 0.01), indicating that the magnitude of sensitization was greater in control mice.

The comparison between groups on day 10 does not provide information about the magnitude of sensitization because the magnitude of sensitization is defined as the difference between the initial response to a drug and the subsequent response to the same dose of the drug. Thus, to compare magnitude of sensitization between control and ESS groups, the change from day one to day 10 between groups (i.e., Con Day 10 - day 1 vs ESS Day 10 -day 1) should be compared. It does appear that the change from the first injection is greater in the ESS group vs Control, but comparing the response on day 10 does not address whether the initial induction of sensitization (i.e., the change in response to the same dose of drug across time) differ between groups. It is fine to point out the day 10 difference, but to assess potential differences in the magnitude of sensitization, the difference from day 1 to day 10 should be plotted as a bar graph and compared between Con and ESS groups.

3) In regards to whether or not differences in CPP are related to learning the authors state, "it is possible that the enhanced CPP observed in the stimulated mice was due to alterations in learning and memory, this is unlikely to be the case given that these mice show deficits in cognitive tasks, such as the object recognition test (Christakis, 2012)." This logic is unclear; just because ESS produces deficits in cognition, that does not mean that it may not also produce learning deficits. Given the substantial amount of work already in this manuscript, it may be best to leave this as an open question for future study by saying the possibility of learning impairments should be investigated in future.

4) Discussion: The discussion of mEPSC frequency increases in the Amygdala and NAc is well done. However, could the authors comment on why they chose these particular sub-regions of the Amygdala (BA) and NAc (shell) and how changes in these regions may (or may not) specifically relate to the behaviors they are interested in (either specifically in this MS or more broadly related to ADHD and addiction)?

5) The authors state, "Given that the BA and NAc are interconnected and can influence circuit function and plasticity (Stuber et al., 2011; Britt et al., 2012), it is likely that the electrophysiological changes that occur in these brain regions following excessive sensory stimulation are contributing to the altered responses to cocaine and previously observed behavioral changes in cognition, novelty-seeking and locomotor activity." The authors should provide specific references showing the involvement of the NAc shell and BA to the behaviors mentioned (cocaine-induced locomotion, novelty-seeking, and locomotor activity in general) or revise the statement accordingly.

Minor Comments:

6) Abstract, "Early life experiences affect the formation of neuronal networks, which can have a profound impact on brain function and behavior." Add in adulthood or later in life.

7) "Together, these findings suggest that excessive sensory stimulation during periods of development significantly alters drug reward and the neural circuits that regulate addiction and attention-deficit hyperactivity disorder." "periods of development" is a bit misleading as only one period of development was manipulated. Consider re-wording.

8) Methods: "All experiments and animal procedures were performed in accordance with the [Author University] animal care committee's regulations and conducted in accordance with the US National Institutes of Health guidelines." There seems to be a typo here.

9) Methods: "Television cartoon shows (e.g., Pokemon, Powerpuff girls, Bakugan) were layered on top of each other and played back to the mice to create an auditory stimulation experience." Perhaps I am not understanding, but this seems to be a visual as well as auditory stimulus.

10) Methods: "In order to better accommodate the higher frequency hearing rage of the mice, different sound tracks were pitch shifted to the higher spectrum of the human hearing range." Why was this normalized to the human hearing range? Perhaps this could be clarified by specifically stating the frequency range used so that others could replicate the procedure.

11) Methods: Please provide information about recording conditions for mIPSCs and how mIPSCs were isolated.

Reviewer #2:

The manuscript has been improved. Most of the changes were toning down statements and acknowledging limitations in the approach. I have no further comments or concerns.
